# N/OFQ modulates orofacial pain induced by tooth movement through CGRP-dependent pathways

**DOI:** 10.1186/s12868-021-00632-5

**Published:** 2021-04-09

**Authors:** Xinyu Yan, Han Han, Shizhen Zhang, Yanzhu Lu, Linghuan Ren, Yufei Tang, Xiaolong Li, Fan Jian, Yan Wang, Hu Long, Wenli Lai

**Affiliations:** Department of Orthodontics, State Key Laboratory of Oral Diseases, National Clinical Center for Oral Research, West China Hospital of Stomatology, Sichuan University, No. 14, Section 3, Ren Min Nan Road, Chengdu, 610041 China

**Keywords:** Orofacial pain, Tooth Movement, Pain, Nociceptin/OFQ, CGRP, Overexpression, Trigeminal Ganglia

## Abstract

**Background:**

Nociceptin/orphanin FQ (N/OFQ) has been revealed to play bidirectional roles in orofacial pain modulation. Calcitonin gene-related peptide (CGRP) is a well-known pro-nociceptive molecule that participates in the modulation of orofacial pain. We aimed to determine the effects of N/OFQ on the modulation of orofacial pain and on the release of CGRP.

**Methods:**

Orofacial pain model was established by ligating springs between incisors and molars in rats for the simulation of tooth movement. The expression level of N/OFQ was determined and pain level was scored in response to orofacial pain. Both agonist and antagonist of N/OFQ receptor were administered to examine their effects on pain and the expression of CGRP in trigeminal ganglia (TG). Moreover, gene therapy based on the overexpression of N/OFQ was delivered to validate the modulatory role of N/OFQ on pain and CGRP expression.

**Results:**

Tooth movement elicited orofacial pain and an elevation in N/OFQ expression. N/OFQ exacerbated orofacial pain and upregulated CGRP expression in TG, while UFP-101 alleviated pain and downregulated CGRP expression. N/OFQ-based gene therapy was successful in overexpressing N/OFQ in TG, which resulted in pain exacerbation and elevation of CGRP expression in TG.

**Conclusions:**

N/OFQ exacerbated orofacial pain possibly through upregulating CGRP.

**Supplementary Information:**

The online version contains supplementary material available at 10.1186/s12868-021-00632-5.

## Background

Orofacial pain induced by tooth movement, highly prevalent among orthodontic patients [1], is characterized by a cascade of inflammatory reactions in periodontal tissues that eventually elicits pain sensation [2]. As is well documented, orofacial pain sensation is initiated at periodontal sensory terminals, modulated at trigeminal ganglia (TG), replayed at trigeminal nucleus and finally reaches sensory cortex via thalamus [2]. Particularly, TG undergo dramatic adaptations in response to orofacial pain [3, 4]. Specifically, abundant proteins are upregulated and downregulated in concert to modulate orofacial pain [5, 6].

Nociceptin/orphanin FQ (N/OFQ) is derived from a precursor, prepronociceptin (PNOC), the sequence of which shares similar structural features to precursors of classical opioid peptides [7]. The receptor of N/OFQ, also designated as ORL1, belongs to the opioid receptor family and is widely distributed in both central and peripheral nervous system, especially abundant in pain modulation regions [8–11]. Unlike other members of opioid family, N/OFQ plays a crucial role in pain modulation in a bidirectional manner, exhibiting either pro- or anti-nociceptive effects, which depends on a series of complex factors including pain quality, administration routes and dosages [12–15]. Our previous study elucidated the pro-nociceptive effect of N/OFQ in periodontal tissue for orofacial pain [16]. However, it is still largely unknown the effects of N/OFQ on orofacial pain in TG.

Calcitonin gene-related peptide (CGRP), a 37-amino-acid neuropeptide, is widely distributed in central and peripheral nervous system [17, 18]. It is well documented that CGRP has an essential role in the modulation of orofacial pain, especially migraine [19, 20]. Our previous study revealed that CGRP played an important role in the modulation of orofacial pain [21]. A further study showed that CGRP was co-expressed with N/OFQ in TG, suggesting their possible interactions in pain modulation [22].

Therefore, in this study, we aimed to explore the specific role of N/OFQ in the modulation of orofacial pain and its impacts on CGRP expression.

## Methods

### Animals

In total, 357 male Sprague–Dawley rats (200–250 g) were obtained from the Animal Experimental Center at Sichuan University (Additional File 1: Table S1). They were maintained in the animal facility and kept in an air-conditioned room at 21 °C with a 12 h light–dark cycle. Standard rat chow and water were provided ad libitum. Animal experiments were performed in accordance with protocols that were approved by the ethical committee of the State Key Laboratory of Oral Diseases, Sichuan University (WCCSIRB-D-2015–006). Following general anesthesia with intraperitoneal injection of sodium pentobarbital (30 mg·kg^−1^), rats were placed in supine positions, and intraoral NiTi alloy closed-coil springs were ligated between left upper first molars and incisors to mimic orthodontic tooth movement. Four initial force levels were used in this study, i.e., 0, 20, 40 and 80 g. Force magnitudes were determined through a force meter (Tiantian, Changsha, China). Rats were euthanized by decapitation following anesthesia with pentobarbital sodium (50 mg·kg^−1^) six hours following drug administrations. Rats that did not receive any intervention were regarded as the baseline control for each group.

### Drug administration into TG

The administration of drugs and lentivirus vectors into TG was conducted according to our previous study with modifications that shaving was not performed to avoid its impact on behavioral testing [23]. Following general anesthesia with intraperitoneal injection of sodium pentobarbital (30 mg·kg^−1^), the injected region was disinfected with 75% ethanol. The injected position is between tympanic bulla and the posterior edge of mandibular ramus. The injected direction is middle upper, perpendicular to the long axis of rat body and 15 degree to coronary plane. When the needle reached trigeminal fossa, the drug was slowly injected at a constant speed. The injection process should last 1 min, and the needle should be remained in situ for 1 min before withdrawn. UFP-101 ([Nphe^1^, Arg^14^, Lys^15^] N/OFQ-NH_2_) is a specific peptide antagonist of N/OFQ which has been reported to have high efficacy and durability in vivo [24]. Specifically, 15 μl nociceptin (10 nM), 15 μl UFP-101 (10 nM) or 15 μl normal saline was administered right after 40-g spring placement (0 h) and on 1st day, 3rd day, 5th day and 7th day to evaluate the effects of N/OFQ on pain and CGRP expression. N/OFQ-overexpressing lentivirus vector suspension (10 μl), blank vector suspension (10 μl) or normal saline (10 μl) was administered into TG one week after spring placement to assess the effects of N/OFQ-based gene therapy on pain.

### Orofacial pain assessment (rat grimace scale, RGS)

**Fig. 1:**
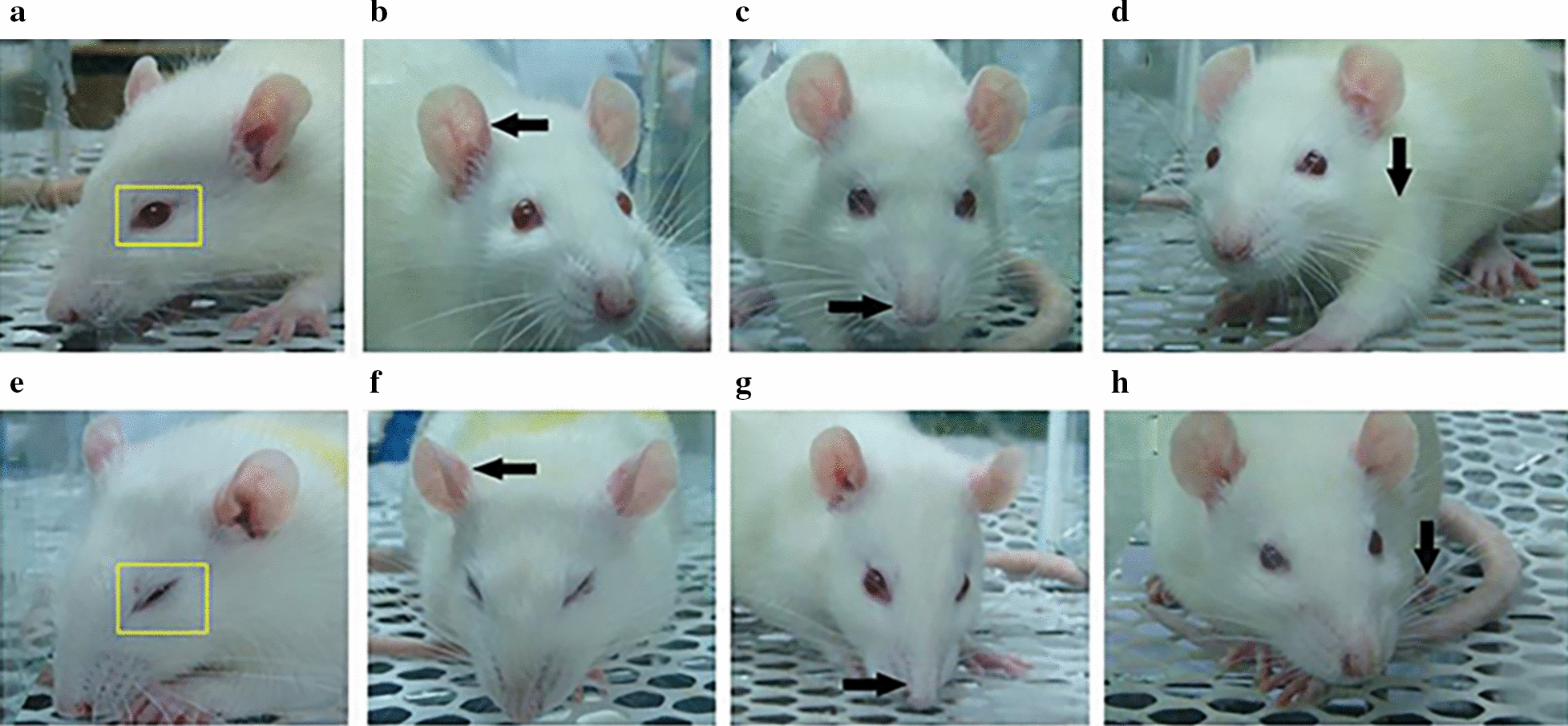
Facial expression changes of rats in RGS scoring. **a**–**d** Facial expression changes in the context of no pain. **e**–**h** Facial expression changes in the context of pain. The RGS scoring was performed based on the facial expression changes in orbit, nose, ear and whisker (indicated by boxes and arrows). Note that the eyes were round, ears were flat, noses were bulged and whiskers showed no tendency of bunching in the context of no pain. In contrast, eyes were squeezed, ears were folded, noses were flattened and whiskers tended to bunch in pain condition

The assessment of orofacial pain was performed through rat grimace scale (RGS) six hours following drug administrations, strictly according to our previous study [25]. In brief, rats were placed in transparent cubicles and videotaped for 30 min using a camera (Canon, Japan). For each session, 10 images of facial expressions for each rat were extracted using an emotional picture capture software (RODENT FACE FINDER). The RGS scoring was performed based on the facial expression changes in orbit, nose, ear and whisker (Fig. 1). Values of 0, 1, or 2 were given according to the degree of the four RGS action units (eyes, ears, nose and whiskers), which was scored in duplicate and independently by two researchers who are blind to the group information and any disagreement was solved by discussion. The scores of four action units were averaged as the score of each photograph, and the mean score of the extracted 10 photographs was calculated as the final RGS score reflecting the level of pain. The surrogate pain levels were obtained by subtracting the baseline RGS scores from the ones for testing sessions.

### Tissue processing and analysis

For immunostaining, V1/V2 of TG were placed in liquid nitrogen and cryosectioned at a thickness of 10 μm by using microtome (Thermo Fisher Scientific, USA). The tissue samples were immunostained with primary antibodies against CGRP (1:200, Ab36001, Abcam, Cambridge, UK) and FITC-labelled rabbit anti-goat secondary polyclonal antibody IgG (1:100, Ab150077, Abcam, Cambridge, UK). Image observation and acquisition were performed using a fluorescence microscope under the 200X lens (Leica, Germany).

For western blot, V1/V2 of TG tissue samples were homogenized through RIPA lysis buffer with PMSF (Beyotime Biotechnology, China). Following electrophoretic separation, proteins were transferred onto PVDF membranes and blocked with 5% skim milk in TBST solution. Afterwards, sealed PVDF membranes were incubated in the primary polyclonal antibody against FLAG (F1804, Sigma, USA), N/OFQ (Ab216413, Abcam, Cambridge, UK) or β-actin (SC-69879, Santa-cruz, USA), then washed with TBST and incubated with secondary polyclonal antibody goat anti-mouse IgG (SC-2005, Santa-cruz, USA). The protein blot densities were analyzed using ImageJ Software (NIH, Bethesda).

Simple Western (WES) analysis was performed on a WES system (ProteinSimple, WS-2471). The V1/V2 of TG tissues were homogenized with RIPA lysis buffer plus PMSF (Beyotime Biotechnology, China). After dilution and degeneration, the extracted proteins, along with the primary polyclonal antibody against CGRP (Ab36001, Abcam, Cambridge, UK), N/OFQ (Ab216413, Abcam, Cambridge, UK) or β-actin (SC-69879, Santa-cruz, USA), horseradish peroxidase (HRP)-conjugated secondary polyclonal antibody IgG (PS-MK15, Protein Simple, USA) and chemiluminescent substrate, were put into the microplate as instructed. The target band intensity of Western Blot results was analyzed using Compass Software. The specificity of antibodies was pre-validated by the antibody manufacturer Abcam (https://www.abcam.com/nociceptin-antibody-ab216413.html). Moreover, in our study, the specificity of primary antibodies was validated according to their corresponding proteins’ specific molecular sizes (20 kD for N/OFQ and 14 kD for CGRP).

Real-time PCR was conducted to determine the mRNA levels of N/OFQ (PNOC gene) and CGRP (Calca gene) with GAPDH being the reference. PCR was performed using specific primers for rat CGRP (forward primer: 5′-GAAGAAGAAGCTCGCCTACTGG-3′, reverse primer: 5′-CTGTCCAAGCTAGAGCCCTCA-3′, expected size: 110 bp) and PNOC (forward primer: 5′-GCTCACGTCCGCTGCTCTTTA-3′, reverse primer: 5′-TCCACCTCATCGGCCTCATCT-3′, expected size: 147 bp). Total RNA was extracted from TG via Trizol RNA Extraction Kit (Pufei, Shanghai, China) and cDNA reversely transcribed through the M-MLV test kit (M1705, Promega). The expressions were quantified in a LightCycler480 (Roche, Switzerland) RT-PCR platform with the SYBR Premix Ex Taq (Takara, Dalian, China). The thermal profile was set at 95 °C for 30 s, followed by 40 cycles at 95 °C for 5 s and then at 60 °C for 30 s.

### Lentivirus vector construction

The lentivirus vector was constructed as previously described [23]. As shown in Fig. 5a, a lentivirus vector GV320 (Shanghai Genechem, China) containing red fluorescence (Cherry) and vector marker (3FLAG) was recombined with rat PNOC gene. PNOC was expressed under ubiquitin promoter, and m-cherry under SV40 promoter. Thus, the two genes were under two independent promoters. The specific sequences retrieved from GenBank (NM_013007) are displayed in Additional file 2. The recombined sequence was amplified with PCR and DNA sequencing performed for sequence verification. Viral vectors were packaged and harvested by transfecting 293 T cells, followed by visualization through fluorescence microscope and verification of PNOC expression through WB. The viral titer was determined to be 2 × 10^8^ TU/ml. All the procedures involving use of lentivirus vectors were performed in the laboratory with biosafety level 2 (BSL2) containment according to General Guidelines for Biosafety in Microbiology and Biomedical Laboratories (WS233-2002).

### Statistical analysis

Results are depicted as mean ± standard error of the mean (SEM). Two-way ANOVA with repeated measures were used to analyze the effects of force magnitudes (0, 20, 40 and 80 g), time (0, 1, 3, 5, 7 and 14 days) and their interactions on RGS scores and the expression levels of N/OFQ. One-way ANOVA with Bonferroni post hoc test was employed to analyze the differences in PNOC expression, CGRP expression and orofacial pain among different time points in each group. Pearson’s correlation test was used to analyze the relevance between N/OFQ expression, CGRP expression and RGS scores. All the statistical analyses were performed in SPSS 19.0 and Graphpad Prism 6.0, with a p value less than 0.05 being considered as statistical significance.

## Results

### Orofacial pain elevated N/OFQ expression in TG

**Fig. 2:**
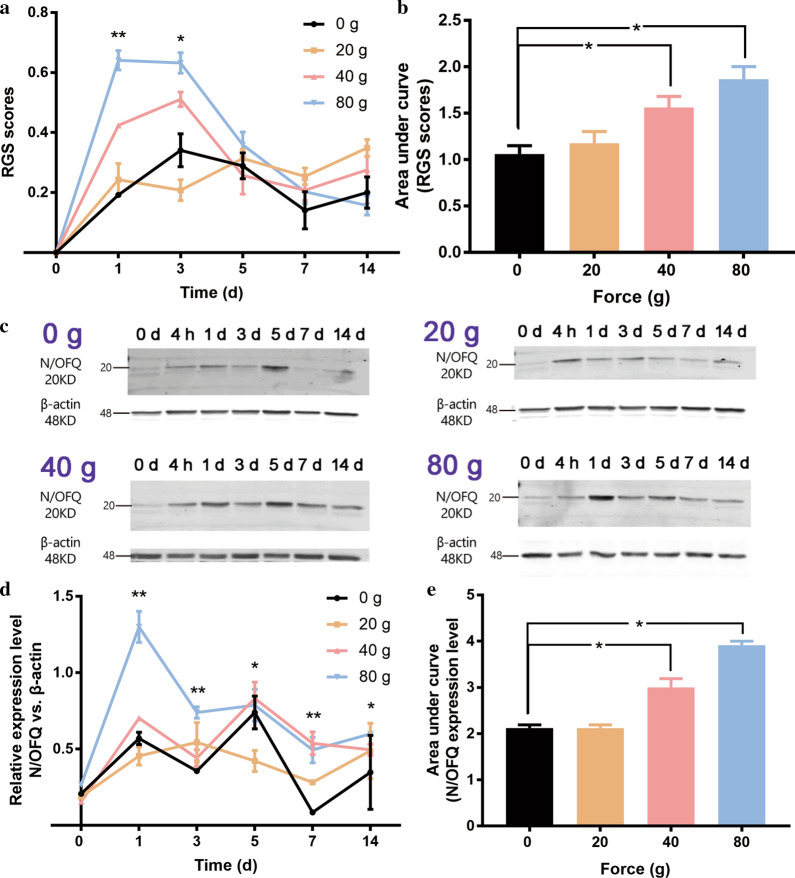
The effect of tooth movement on orofacial pain level and N/OFQ expression. The rats were respectively treated with the force of 0 g, 20 g, 40 g and 80 g (n = 30 for each group, n = 5 for each group each day). **a** Temporal changes of RGS scores in all the force groups. Two-way ANOVA with repeated measures were used to analyze the effects of force magnitudes, time and their interactions on the RGS scores. One-way ANOVA was used to analyze RGS scores in different force groups at different timepoint (*p < 0.05, **p < 0.01, 0 g group vs. 20 g group vs. 40 g group vs. 80 g group). **b** One-way ANOVA with Bonferroni post hoc test was used to analyze the area under curve (AUC) of RGS scores (*p < 0.05, 40 g group vs. 0 g group, 80 g group vs. 0 g group). **c** Western blot analysis for the quantification of N/OFQ expression in trigeminal ganglia, with β-actin being the internal reference. The images were cropped using Adobe Photoshop software, and full-length blots/gels are presented in Additional file 3: Figure S1. **d** Temporal changes of N/OFQ expression in all the force groups. Two-way ANOVA with repeated measures were used to analyze the effects of force magnitudes, time and their interactions on the expression levels of N/OFQ. One-way ANOVA was used to analyze N/OFQ expression levels in different force groups at the same timepoint (*p < 0.05, **p < 0.01, 0 g group vs. 20 g group vs. 40 g group vs. 80 g group). **e** One-way ANOVA with Bonferroni post hoc test was used to analyze the AUC of the expression level of N/OFQ (*P < 0.05, 40 g group vs. 0 g group, 80 g group vs. 0 g group)

We found that orofacial pain-like behaviors indicated by RGS scores was elicited following tooth movement and started to increase on 1st day, peaked on 3rd day, decreased on 5th day and returned to baseline level on 7th or 14th day (Fig. 2a). The two-way ANOVA with repeated measures revealed that RGS scores were significantly influenced by time (p < 0.001), force magnitude (p = 0.005) and their interactions (p < 0.001). The one-way ANOVA revealed that RGS scores were significantly different on 1st day (p < 0.01) and 3rd day (p < 0.05). Areas under curve (AUCs) were similar between 0-g group and 20-g group (p > 0.05), while significantly higher in the 40-g group (p < 0.05) and 80-g group (p = 0.001) (Fig. 2b).

As displayed in Fig. 2c, d, our results revealed that the expression levels of N/OFQ started to increase on 1st day following orthodontic tooth movement. The one-way ANOVA revealed that N/OFQ expression was significantly different on 1st day (p < 0.01), 3rd day (p < 0.01), 5th day (p < 0.05), 7th day (p < 0.01) and 14 day (p < 0.05). Areas under curve (AUCs) were similar between 0-g group and 20-g group (p > 0.05), while significantly higher in the 40-g group (p < 0.001) and 80-g group (p = 0.001) (Fig. 2e). Thus, a force magnitude of 40 g was used for the following experiments.

### The effects of N/OFQ agonist and antagonist on pain modulation and CGRP expression

**Fig. 3:**
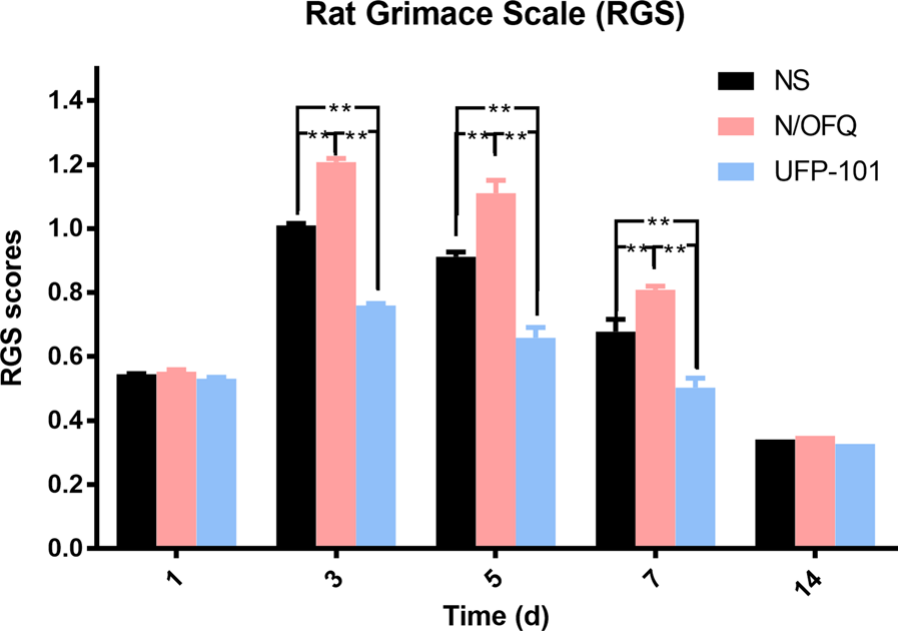
The effects of N/OFQ receptor agonist and antagonist on pain modulation. The rats were respectively injected with N/OFQ receptor agonist (N/OFQ), antagonist (UFP-101) and normal saline (NS) after 40-g spring placement (n = 8 for each group). Temporal changes of RGS scores in NS group, N/OFQ group and UFP-101 group were shown in the figure. One-way ANOVA with Bonferroni post hoc test was employed to analyze the effects of N/OFQ, UFP-101 and NS on RGS scores. (**p < 0.01, NS group vs. N/OFQ group, NS group vs. UFP-101 group, N/OFQ group vs. UFP-101 group)

As depicted in Fig. 3, two-way ANOVA with repeated measures indicated that the RGS scores were significantly influenced by different groups (p < 0.05) and time (p < 0.05). The RGS scores were significantly higher in the N/OFQ agonist group than in the normal saline (NS) group on 3rd day, 5th day and 7th day (p < 0.01). Moreover, the RGS scores were significantly lower in the antagonist UFP-101 group than in the NS group on 3rd day, 5th day and 7th day (p < 0.01).

**Fig. 4:**
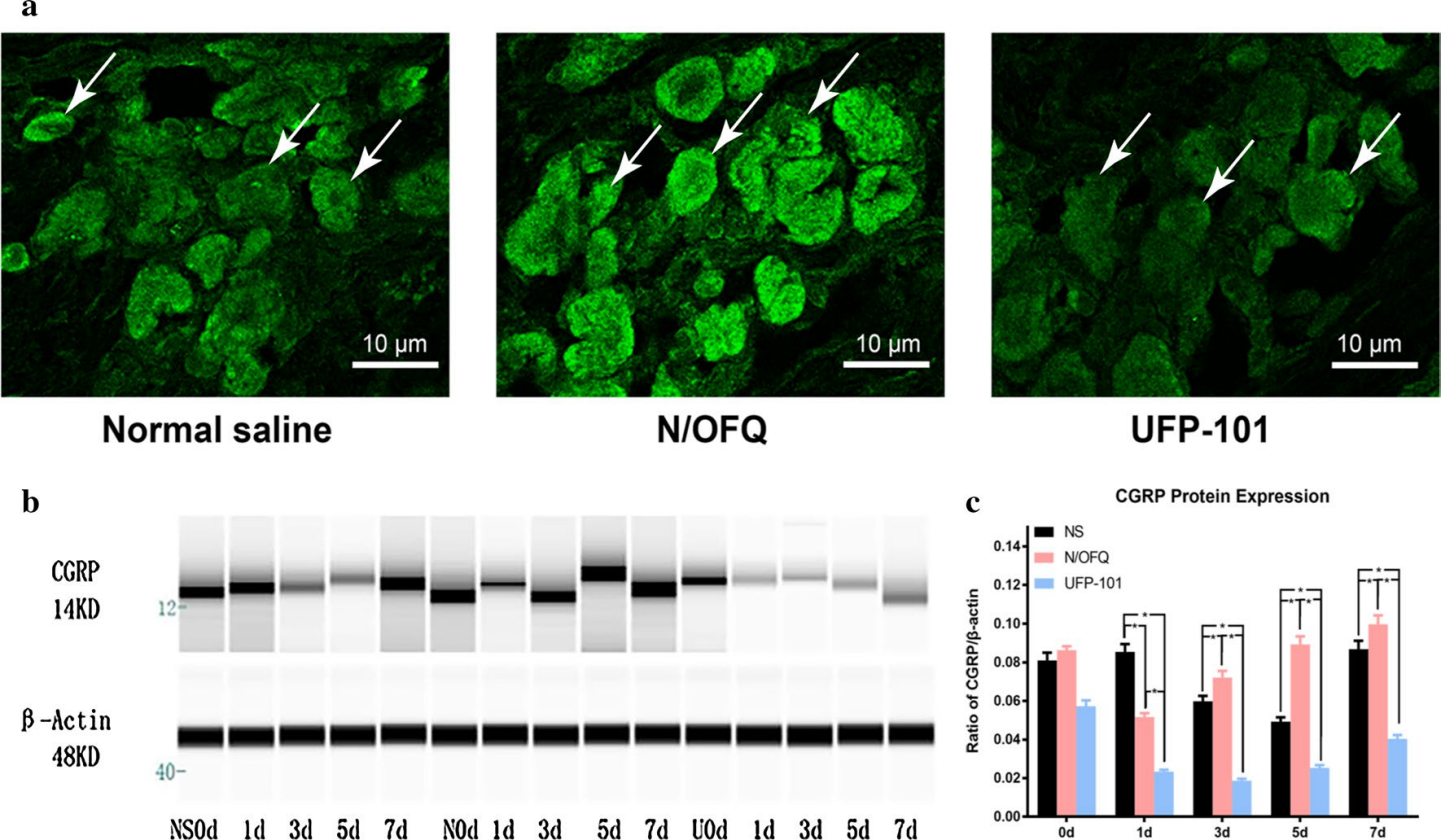
The effects of N/OFQ receptor agonist and antagonist on CGRP expressions. The rats were respectively injected with N/OFQ receptor agonist (N/OFQ), antagonist (UFP-101) and normal saline (NS) after 40-g spring placement (n = 18 for each group, n = 3 for each group each day). **a** CGRP immunostaining in trigeminal ganglion of rats in NS group, N/OFQ group and UFP-101 group (3rd day). The images were acquired at the resolution of 300dpi. **b** Western blot analysis of CGRP expression in trigeminal ganglion of rats in NS group, N/OFQ group and UFP-101 group using WES. The images were cropped using Adobe Photoshop software, and full-length blots/gels are presented in Additional file 3: Figure S2. **c** Temporal changes of CGRP expression level in NS group, N/OFQ group and UFP-101 group. One-way ANOVA with Bonferroni post hoc test was employed to analyze the effects of N/OFQ, UFP-101 and NS on CGRP expressions. (*p < 0.05, NS group vs. N/OFQ group, NS group vs. UFP-101 group, N/OFQ group vs. UFP-101 group)

As displayed in Fig. 4a and b, more CGRP-positive neurons were detected in the N/OFQ group and fewer CGRP-positive neurons were observed in the UFP-101 group. Two-way ANOVA with repeated measures indicated that CGRP expression was significantly influenced by different groups (p < 0.05) and time (p < 0.05). The expression levels of CGRP were significantly higher in the N/OFQ group than in the NS group on 3rd day, 5th day and 7th day (all p < 0.05), and significantly lower in the UFP-101 group than in the NS group on 1st day, 3rd day, 5th day and 7th day (all p < 0.05) (Fig. 4c).

### Successful transduction of PNOC-overexpressing lentivirus vectors into TG

**Fig. 5:**
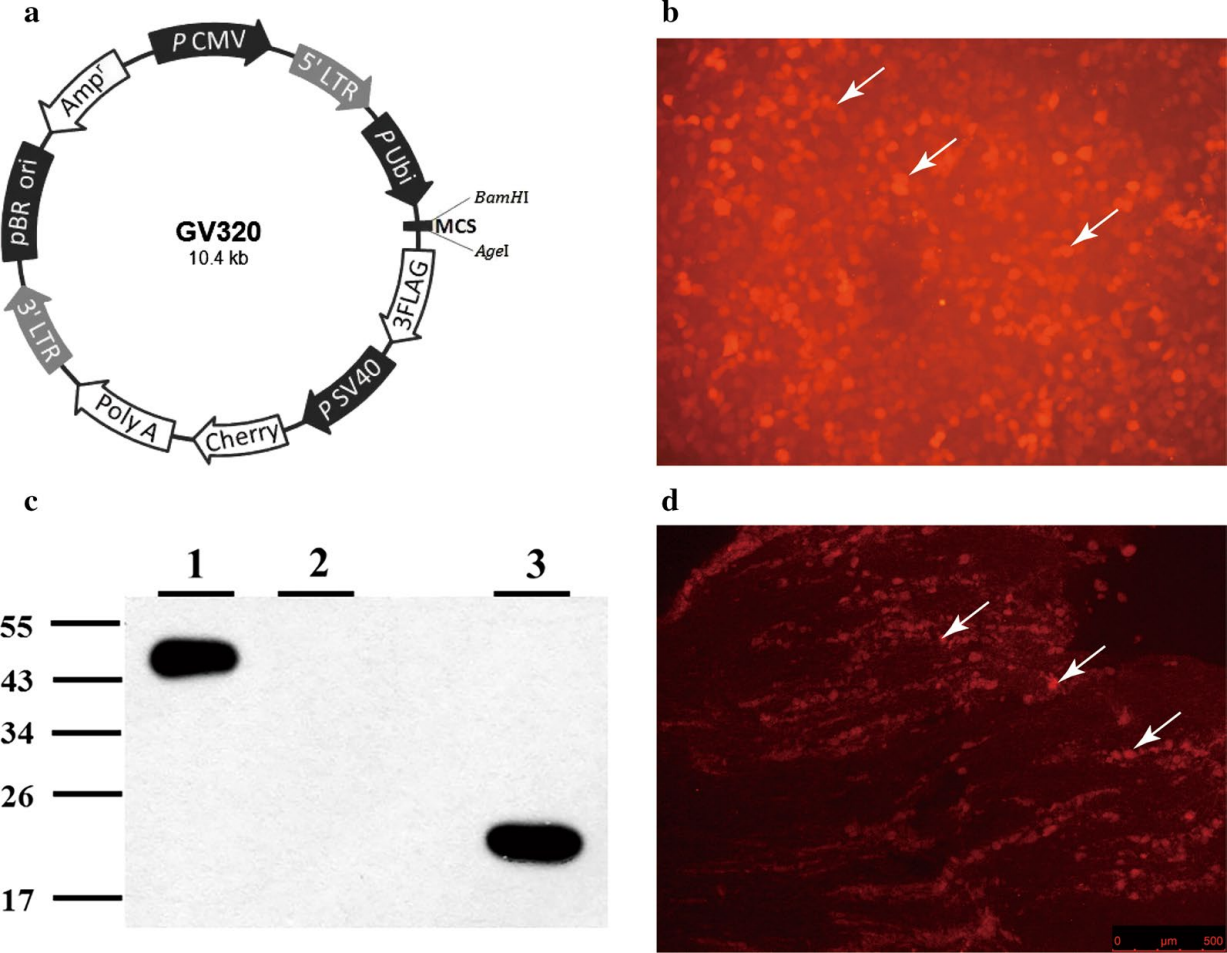
Construction of PNOC overexpression lentivirus vector. **a** The map of GV320 vector. **b** Successful transduction of lentiviral vector into 293 T cells as shown by fluorescence microscopy that most cells were marked by enhanced red fluorescence protein (Cherry). The image was acquired at the resolution of 300dpi. **c** Western blot analysis of 293 T cells transfected with positive reference (SURVIVIN-3FLAG-GFP, 48KD) [1], not transfected [2], and transfected with PNOC lentiviral vectors (N/OFQ-3FLAG, 23KD) [3]. **d** Immunofluorescence image of TG in rat stained with cherry, which indicates successful transfection with PNOC lentivirus. The image was acquired at the resolution of 300dpi

As shown in Fig. 5a, the lentivirus vector carried cherry fluorescence protein and we used this fluorescence signal for the detection of virus transduction. As displayed in Fig. 5b, the PNOC-overexpressing lentivirus vectors were able to transduce 293 T cells, as evidenced by the cherry fluorescence. This overexpression was functionally successful as the N/OFQ-3FLAG fusion protein was detected (Fig. 5c). Moreover, immunofluorescence staining showed that lentivirus vectors were transduced at trigeminal ganglia (Fig. 5d).

**Fig. 6:**
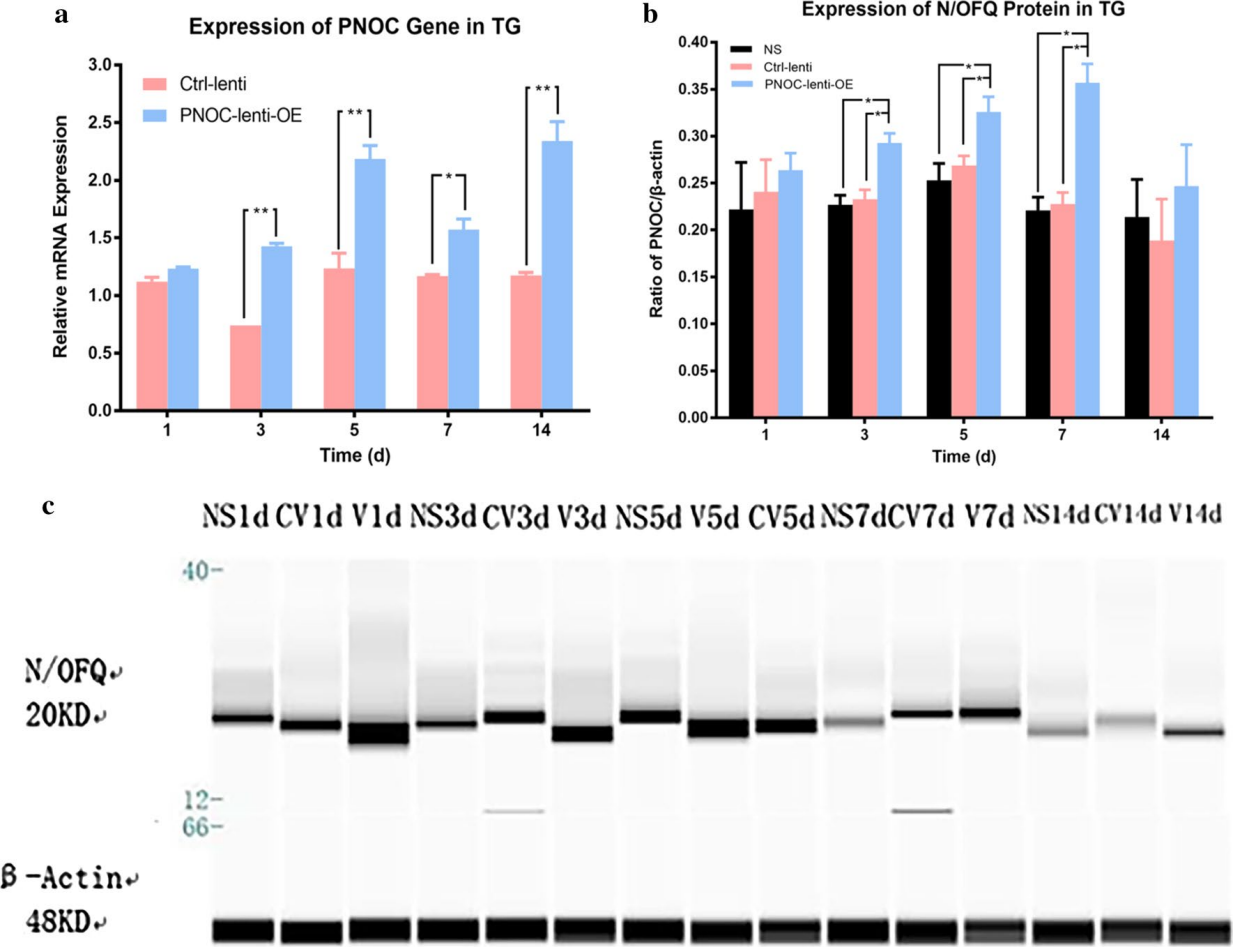
The effects of PNOC overexpression lentivirus vector on PNOC and N/OFQ expression. The rats were respectively administered with PNOC overexpression lentivirus (PNOC-lenti-OE), control lentivirus (ctrl-lenti) and normal saline (NS) one week prior to 40-g force application (n = 15 for each group, n = 3 for each group each day). **a** Temporal changes of PNOC mRNA expression level in trigeminal ganglia of rats in PNOC lentivirus group and control lentivirus group. One-way ANOVA with Bonferroni post hoc test was employed to analyze the effects of control lentivirus and PNOC overexpression lentivirus on PNOC mRNA expression. (*p < 0.05, **p < 0.01, ctrl-lenti group vs. PNOC-lenti-OE group). **b** Temporal changes of N/OFQ expression in trigeminal ganglia of rats in NS group, control lentivirus group and PNOC lentivirus group. One-way ANOVA with Bonferroni post hoc test was employed to analyze the effects of normal saline, control lentivirus and PNOC overexpression lentivirus on N/OFQ expression. (*p < 0.05, PNOC-lenti-OE group vs. NS group, PNOC-lenti-OE group vs. ctrl-lenti group). **c** Western blot analysis of N/OFQ expression in trigeminal ganglion of rats in NS group, control lentivirus group and PNOC lentivirus group using WES (*NS* normal saline, *CV* control lentivirus, *V* PNOC lentivirus). The images were cropped using Adobe Photoshop software, and full-length blots/gels are presented in Additional file 3: Figure S3

Two-way ANOVA with repeated measures indicated that the expression level of both PNOC gene and N/OFQ protein were significantly influenced by different groups (p < 0.05) and time (p < 0.05). The real-time PCR revealed that the expression level of PNOC gene was significantly higher in the PNOC-lenti-OE group (denoting PNOC-overexpressing lentivirus transduction) than in the Ctrl-lenti group (blank lentivirus vector) on 3rd day, 5th day, 7th day and 14th day (all p < 0.05) (Fig. 6a). The western blot showed that the level of N/OFQ protein was significantly higher in the PNOC-lenti-OE group than in the Ctrl-lenti group and the NS group on 3rd day, 5th day and 7th day (all p < 0.05) (Fig. 6b, c).

### The effects of N/OFQ overexpression on CGRP expression and pain modulation

**Fig. 7:**
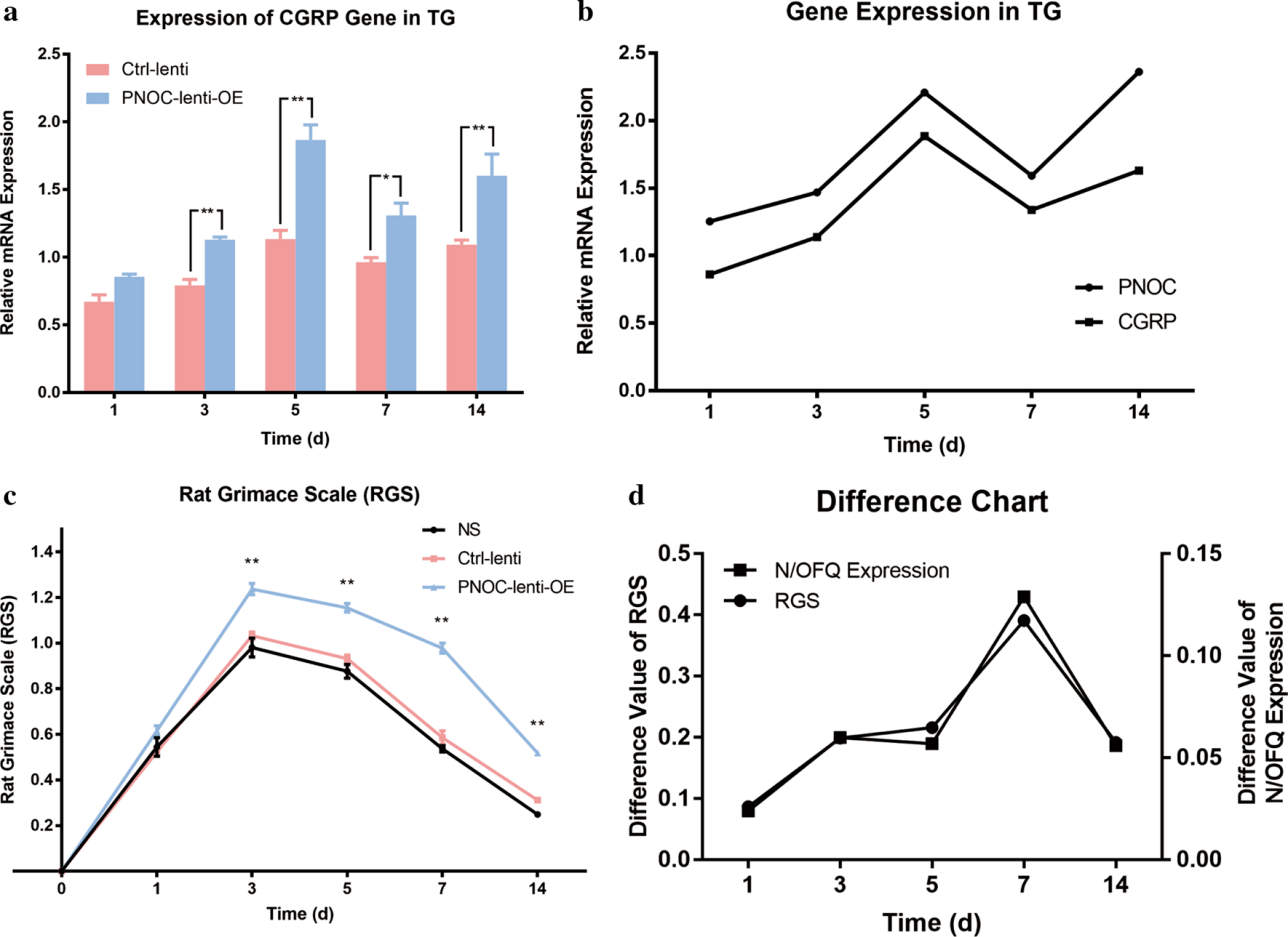
The effects of PNOC overexpression on CGRP expressions and pain modulation. The administration of lentivirus or saline was performed one week prior to force application (40 g). **a** Temporal changes of CGRP mRNA expression in trigeminal ganglia in control lentivirus group and PNOC lentivirus group (n = 15, n = 3 for each group each day). One-way ANOVA with Bonferroni post hoc test was employed to analyze the effects of control lentivirus and PNOC overexpression lentivirus on CGRP mRNA expression. (*p < 0.05, **p < 0.01, ctrl-lenti group vs. PNOC-lenti-OE group). **b** Changes in PNOC expression and CGRP expression in PNOC lentivirus group exhibited high correlation (r = 0.925). **c** Temporal changes of RGS scores in NS group, control lentivirus group and PNOC lentivirus group (n = 8 for each group). One-way ANOVA with Bonferroni post hoc test was employed to analyze the effects of normal saline, control lentivirus and PNOC overexpression lentivirus on RGS scores. (**p < 0.01, PNOC-lenti-OE group vs. NS group, PNOC-lenti-OE group vs. ctrl-lenti group). **d** Difference value of orofacial pain level and N/OFQ expression level between PNOC lentivirus group and control lentivirus group. The trends of the two curves are similar and highly correlated (r = 0.994)

As displayed in Fig. 7a, two-way ANOVA with repeated measures indicated that the CGRP expression was significantly influenced by different groups (p < 0.05) and time (p < 0.05). The expression level of CGRP was significantly higher in the PNOC-lenti-OE group than in the Ctrl-lenti group on 3rd day, 5th day, 7th day and 14th day (all p < 0.05). Moreover, as depicted in Fig. 7b, the trend of the changes in expressions of CGRP and N/OFQ were highly positively correlated (r = 0.925).

RGS scoring revealed that pain levels were significantly higher in the PNOC-lenti-OE group than in the Ctrl-lenti group and the NS group on 3rd day, 5th day, 7th day and 14th day (all p < 0.01) (Fig. 7c). Then, we plotted the difference values between PNOC-lenti-OE and Ctrl-lenti groups against time for RGS scores and N/OFQ expression level. We found that the changes in difference values of RGS scores and N/OFQ expression level displayed high consistency which were most prominent on 7th day. Pearson’s correlation test showed that the trend of the difference values for RGS scores and N/OFQ expression level were highly positively correlated (r = 0.994) (Fig. 7d).

## Discussion

In this study, we found that N/OFQ expression was elevated in response to orofacial pain elicited by tooth movement. N/OFQ could exacerbate orofacial pain and elevate CGRP expressions while UFP-101 alleviated orofacial pain and downregulate CGRP expressions. Furthermore, overexpression of PNOC gene that upregulated N/OFQ in trigeminal ganglia was able to exacerbate pain level and increase CGRP expression level in trigeminal ganglia.

Orofacial pain, a constellation of various painful conditions in orofacial regions, includes migraine, trigeminal neuralgia, headaches, dental pain and tooth-movement pain [26, 27]. Of particular, orofacial pain induced by tooth movement is a type of inflammatory pain at periodontal tissues due to force application [25]. It is well accepted that tooth movement elicits orofacial pain by obstructing periodontal blood vessels that in turn initiate a cascade of inflammatory response [28]. In the present study, we used NiTi-closed coil springs to elicit tooth-movement-related orofacial pain. Notably, several studies have suggested that constant forces cannot be delivered by NiTi-closed coil springs, which could be partially attributed to the tooth movement that changed the length of the springs. Thus, it is more appropriate to state “initial force”. On the whole, however, this tooth-movement animal model has been well-documented and validated by many previous studies, including ours [28–30]. We previously revealed that tooth-movement-induced orofacial pain was initiated by a force above 20 g and that pain level differed among rats receiving different force magnitudes [2]. But it is still largely unknown whether this paradigm works similarly between force magnitude and the expression level of N/OFQ in TG. Notably, we found that pain level and N/OFQ expression level were upregulated in the 0-g group, which could be attributed to the painful stimulus induced by bulky intraoral springs [25]. Furthermore, the AUCs of pain and N/OFQ were similar between the 0-g group and the 20-g group, while significantly higher in the 40-g group and 80-g group, indicating that the threshold force that could incite the tooth-movement-dependent upregulation of pain level and N/OFQ lies between 20 and 40 g, which is consistent with our previous study [25]. Therefore, we suggest that 40-g force could be sufficient to elicit orofacial pain and an elevation of N/OFQ expression in trigeminal ganglia.

N/OFQ and its receptor are the fourth opioid family member discovered so far [31]. Despite high sequence similarity between N/OFQ and other opioid ligands, the lacking of an N-terminal tyrosine renders N/OFQ to have a negligible affinity for the three classical opioid receptors, thereby making it functionally distinct from the classical opioid ligands (e.g., morphine) [32]. Though NOP receptor inhibits voltage-gated calcium channels and activates inward potassium channels coupled to pertussis toxin-sensitive Gi/o proteins, thereby impacting on the neurotransmitter release and neuronal excitability like other opioid receptors, the pain-modulatory effects mediated by N/OFQ-NOP are more complicated [31]. N/OFQ has been revealed to exhibit either pro- or anti-nociceptive effects, depending on a series of complex factors such as pain quality, doses and administration routes [12–15]. In terms of rodents, N/OFQ system exhibits antinociceptive effects when peripherally and spinally activated, while pronociceptive effects after supraspinal activation. Therefore, the net effect of systemically administered NOP agonists on nociception is dependent on relative contribution of peripheral, spinal and supraspinal sites of action, which varies between rodents and non-human primates [33]. The sensation of orofacial pain induced by tooth movement is initially received by the sensory terminals at periodontium, transmitted to TG, relayed to trigeminal nucleus at medulla oblongata and projected to sensory cortex via thalamus [2]. Our previous studies have reported that N/OFQ participates in pain modulation at periodontium and trigeminal nucleus caudalis [16, 34]. Although previous findings have demonstrated that N/OFQ and its receptor are actively expressed in neuronal cells of trigeminal ganglia and our previous study found an indirect evidence that N/OFQ could modulate the expression of a key nociceptor (P2X3) on trigeminal neurons [22, 35], its pain-modulatory role at TG is still largely unknown. Our results revealed that intra-ganglionic administration of N/OFQ exacerbated pain while that of UFP-101 alleviated pain, suggesting that N/OFQ plays a pro-nociceptive role in orofacial pain induced by tooth movement. Moreover, elevated pain level caused by PNOC overexpression lentivirus was strongly correlated with increased N/OFQ expression, strongly supporting the promotive role of N/OFQ in pain modulation. This finding was consistent with our previous studies where we found that periodontal administration of N/OFQ antagonist UFP-101 was able to alleviate pain in rats and that N/OFQ could upregulate the expression of a nociceptor P2X3 [16, 35]. However, Borgland et al. revealed that N/OFQ inhibited calcium currents of trigeminal neurons, supporting its antinociceptive role in TG [36]. We attribute this disagreement to the fact that N/OFQ was able to inhibit calcium currents in only a subpopulation of trigeminal neurons in the study by Borgland et al. [36].

CGRP is a well-known pronociceptive molecule for orofacial pain [37]. The upregulation and release of CGRP are the hallmarks during pain episodes of orofacial pain [38]. The release of CGRP to peripheral tissues could induce neurogenic inflammation that exacerbates pain [39, 40]. And we found CGRP was upregulated and released to periodontal tissues in response to orofacial pain in our previous studies [21, 41, 42]. However, current evidence on the interaction between CGRP and N/OFQ is generally lacking, especially in the aspect of pain modulation. Previous studies have shown that N/OFQ were abundantly expressed with a high degree of coexpression with CGRP both in dorsal root ganglion and trigeminal ganglion, suggesting that N/OFQ modulated primary sensory nociception through interacting with CGRP [22, 43]. In trigeminal neurons, N/OFQ-NOP system has been reported to impact on stimulated CGRP release, whereas how N/OFQ interacts with CGRP in response to pain stimuli is largely unknown [44, 45]. In our present study, we found that N/OFQ upregulated while UFP-101 downregulated the expression level of CGRP in trigeminal ganglia, supporting the notion that N/OFQ has a pronociceptive role in trigeminal ganglia via promoting CGRP expression and release, thus facilitating transmission of pain signals. Noteworthily, UFP-101 actively decreased the amount of CGRP protein by approximately 60% on 1st day, which was attributed to the accumulative effect of UFP-101 administered respectively at baseline and 1st day.

In terms of methodology of this study, facial expressions have been widely used to evaluate pain level of non-verbal experimental animals, among which RGS is a standardized behavioral coding system demonstrated to have high accuracy and reliability [46]. Interestingly, the RGS scores were greater for the NS group in Fig. 3 than that in 40-g group of Fig. 2a since 3rd day, which can be mainly attributed to the fact that the rats of Fig. 3 received drug injection that aggravated their pain level, thus leading to generally higher RGS scores. The integration of viral gene into host genome on one hand is beneficial for stable expression, which makes it a promising gene therapeutic tool for a variety of pain conditions, especially for chronic pain [47–49]. Our previous study revealed that administration of lentivirus containing shRNA aiming at knocking down TRPV1 or ASIC3 was effective in alleviating orofacial pain [50, 51]. In our present study, transduction of PNOC overexpression lentivirus was successful in overexpressing N/OFQ in trigeminal ganglia and the overexpression was stable on 3rd day, 5th day and 7th day, but not on 14th day. We attribute this decrease of N/OFQ expression on 14th day to built-in analgesic pathways that were activated at the late stage of orofacial pain. Notably, the increase in N/OFQ did not reach statistical significance on 1st day (Fig. 6b), which might be significantly different if the virus was injected earlier (e.g., two weeks before spring placement). We showed that the overexpression of N/OFQ was able to upregulate CGRP expression and exacerbate orofacial pain induced by tooth movement. Moreover, the N/OFQ expression levels and pain levels were highly correlated with each other, further reinforcing the aforementioned results. Notably in Fig. 4c, the expression level of CGRP in NS group was slightly increased on 1st day, but significantly decreased on 3rd and 5th day, suggesting that trigeminal injection inhibited CGRP expression in TG. In contrast, the expression level of CGRP was significantly higher in N/OFQ group, which revealed that N/OFQ upregulated CGRP expression in TG.

Though our study firstly revealed the modulatory role of N/OFQ system in tooth-movement-induced orofacial pain which is dependent on CGRP, there are some limitations in this study. Although CGRP has been well-documented as a pronociceptive factor in TG neural system, and our previous study has validated the pain-modulatory effect of CGRP on tooth-movement-induced pain, direct evidence on the regulatory role of CGRP on tooth-movement-induced pain in our present study was not validated. Besides, while gain-of-function study conducted using PNOC overexpression lentivirus suggested the pro-nociceptive effect of N/OFQ in trigeminal ganglia, the knockdown of PNOC may be more important to validate the therapeutic value of N/OFQ. Moreover, the role of NOP receptor and its relationship with orofacial pain and CGRP expression need specific investigations. Therefore, based on above limitations, further studies digging into more thorough pain-modulatory mechanisms of N/OFQ-NOP system are expected.

## Conclusions

Taken together, we suggest that N/OFQ modulate orofacial pain induced by tooth movement possibly through CGRP-dependent pathways.

## Supplementary Information

**Additional file 1: Table S1.** Grouping of experimental animals.

**Additional file 2:** Rat PNOC overexpression sequence. The specific sequences were retrieved from GenBank (NM_013007), with red indicating PNOC mRNA sequence, and ACCGGT is AgeI enzyme cutting site.

**Additional file 3: Figure S1.** Full results of Western blot analysis for the quantification of N/OFQ expression in trigeminal ganglia. **Figure S2.** Full results of Western blot analysis of CGRP expression in trigeminal ganglion of rats in NS group, N/OFQ group and UFP-101 group. **Figure S3.** Full results of Western blot analysis of N/OFQ expression in trigeminal ganglion of rats in NS group, control lentivirus group and PNOC lentivirus group.

## Data Availability

All data generated or analysed during this study are included in this published article and its supplementary information files.
