# Supplementary material for: N/OFQ modulates orofacial pain induced by tooth movement through CGRP-dependent pathways
Source: BMC Neurosci. 2021 Apr 9;22:25. doi: 10.1186/s12868-021-00632-5 (PMC8034138; doi:10.1186/s12868-021-00632-5)
Supplement: Supplementary file 1 — Additional file 1: Table S1. Grouping of experimental animals. [file 12868_2021_632_MOESM1_ESM.docx]

**Additional File 1. Grouping of experimental animals.**

| Experiment | Grouping | Number of animals |
| --- | --- | --- |
| RGS scoring | 0-g group | 30 |
|  | 20-g group | 30 |
|  | 40-g group | 30 |
|  | 80-g group | 30 |
|  | Normal saline (NS) group | 8 |
|  | N/OFQ group | 8 |
|  | UFP-101 group | 8 |
|  | Normal saline (NS) group | 8 |
|  | Control lentivirus (Ctrl-lenti) group | 8 |
|  | PNOC overexpression lentivirus (PNOC-lenti-OE) group | 8 |
| Immunofluorescence analysis of CGRP protein expression | Normal saline (NS) group | 18 |
|  | N/OFQ group | 18 |
|  | UFP-101 group | 18 |
| WES analysis of CGRP protein expression | Normal saline (NS) group | 15 |
|  | N/OFQ group | 15 |
|  | UFP-101 group | 15 |
| WES analysis of N/OFQ protein expression | Normal saline (NS) | 15 |
|  | Control lentivirus (Ctrl-lenti) | 15 |
|  | PNOC overexpression lentivirus (PNOC-lenti-OE) | 15 |
| RT-qPCR of PNOC and CGRP gene expression | Normal saline (NS) | 15 |
