# Supplementary material for: N/OFQ modulates orofacial pain induced by tooth movement through CGRP-dependent pathways
Source: BMC Neurosci. 2021 Apr 9;22:25. doi: 10.1186/s12868-021-00632-5 (PMC8034138; doi:10.1186/s12868-021-00632-5)
Supplement: Supplementary file 2 — Additional file 2: Rat PNOC overexpression sequence. The specific sequences were retrieved from GenBank (NM_013007), with red indicating PNOC mRNA sequence, and ACCGGT is AgeI enzyme cutting site. [file 12868_2021_632_MOESM2_ESM.docx]

**Additional File 2. Rat Pnoc overexpression sequence**

ACCGGTCGCCACCATGAAAATCCTGTTTTGTGATGTCCTGCTGCTCAGCCTGCTCTCCAGCGTGTTCAGCAGCTGTCCCGAGGACTGCCTCACCTGCCAGGAGAGGCTCCACCCGGCTCCGGGCAGCTTCAACCTGAAGCTGTGCATCCTCCAGTGTGAAGAGAAGGTCTTCCCCCGCCCTCTCTGGACTCTTTGCACCAAAGCCATGGCCAGTGACTCTGAGCAGCTCAGCCCTGCTGATCCAGAGCTCACGTCCGCTGCTCTTTACCAGTCGAAAGCCTCGGAGATGCAGCACCTGAAGAGAATGCCGCGTGTCAGGAGTGTGGTGCAAGCCCGAGACGCAGAGCCTGAGGCAGATGCAGAGCCTGTCGCAGATGAGGCCGATGAGGTGGAGCAGAAGCAGCTGCAGAAAAGGTTTGGGGGCTTCACTGGGGCCCGGAAGTCAGCCCGGAAGTTGGCCAACCAGAAGCGGTTCAGTGAGTTTATGAGGCAGTACCTGGTCCTGAGCATGCAGTCAAGCCAACGCCGGCGCACTCTGCACCAGAATGGTAATGTGACCGGT

(Red indicates PNOC mRNA sequence, ACCGGT is AgeI enzyme cutting site)
