# Supplementary material for: N/OFQ modulates orofacial pain induced by tooth movement through CGRP-dependent pathways
Source: BMC Neurosci. 2021 Apr 9;22:25. doi: 10.1186/s12868-021-00632-5 (PMC8034138; doi:10.1186/s12868-021-00632-5)

**Supplementary Figure 1 (matched Figure 2C)**


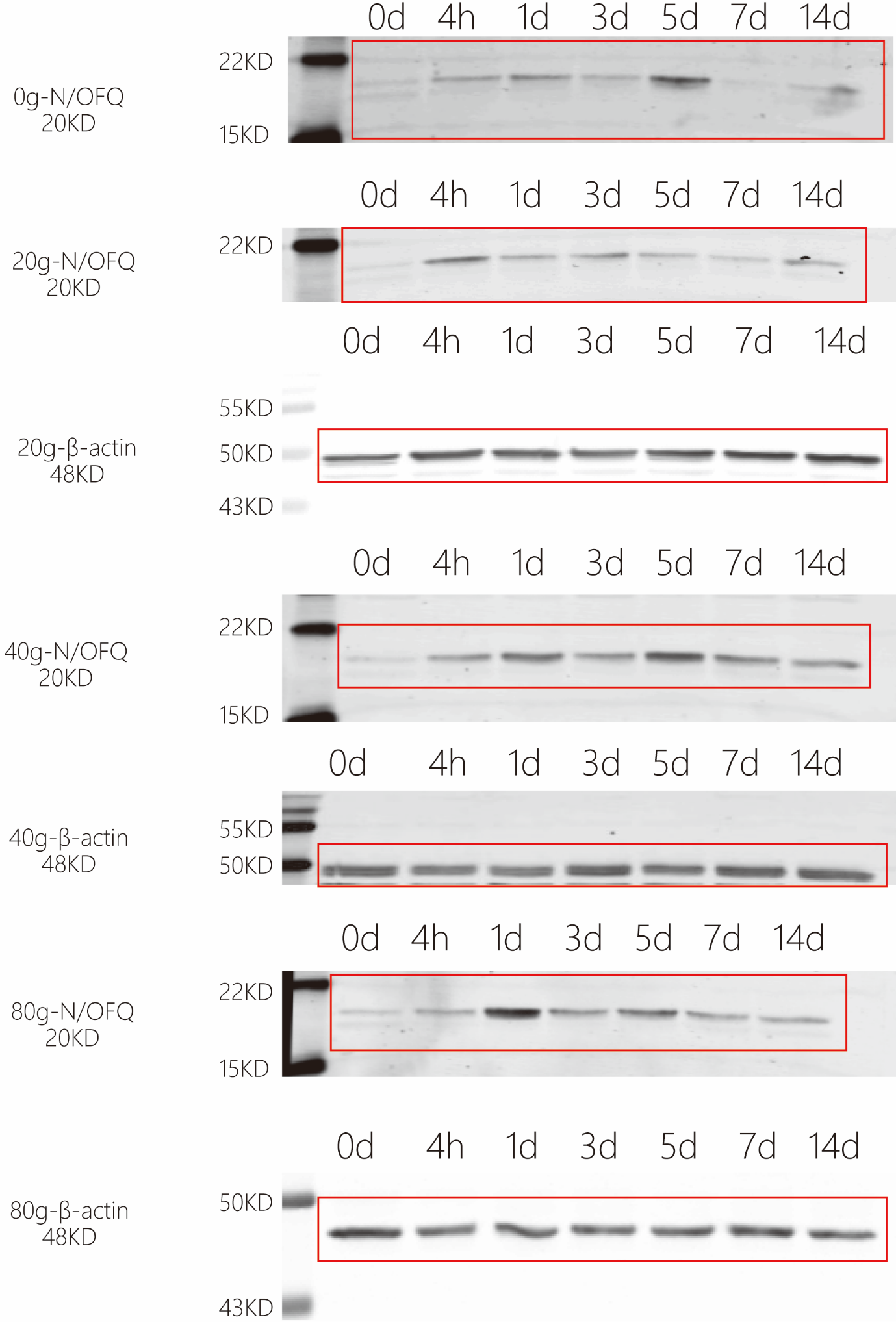


**Supplementary Figure 2 (matched Figure 4B)**


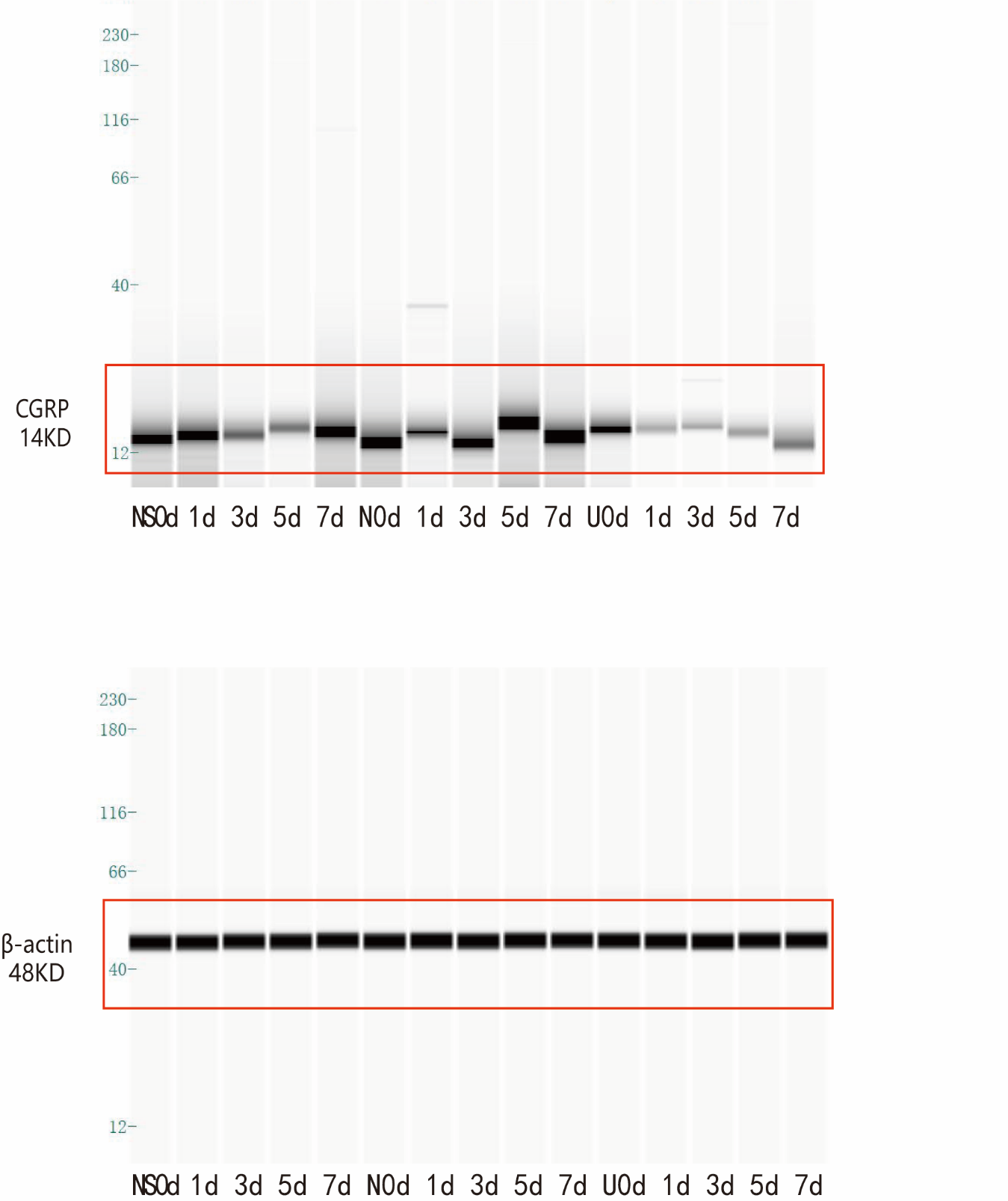


**Supplementary Figure 3 (matched Figure 6C)**


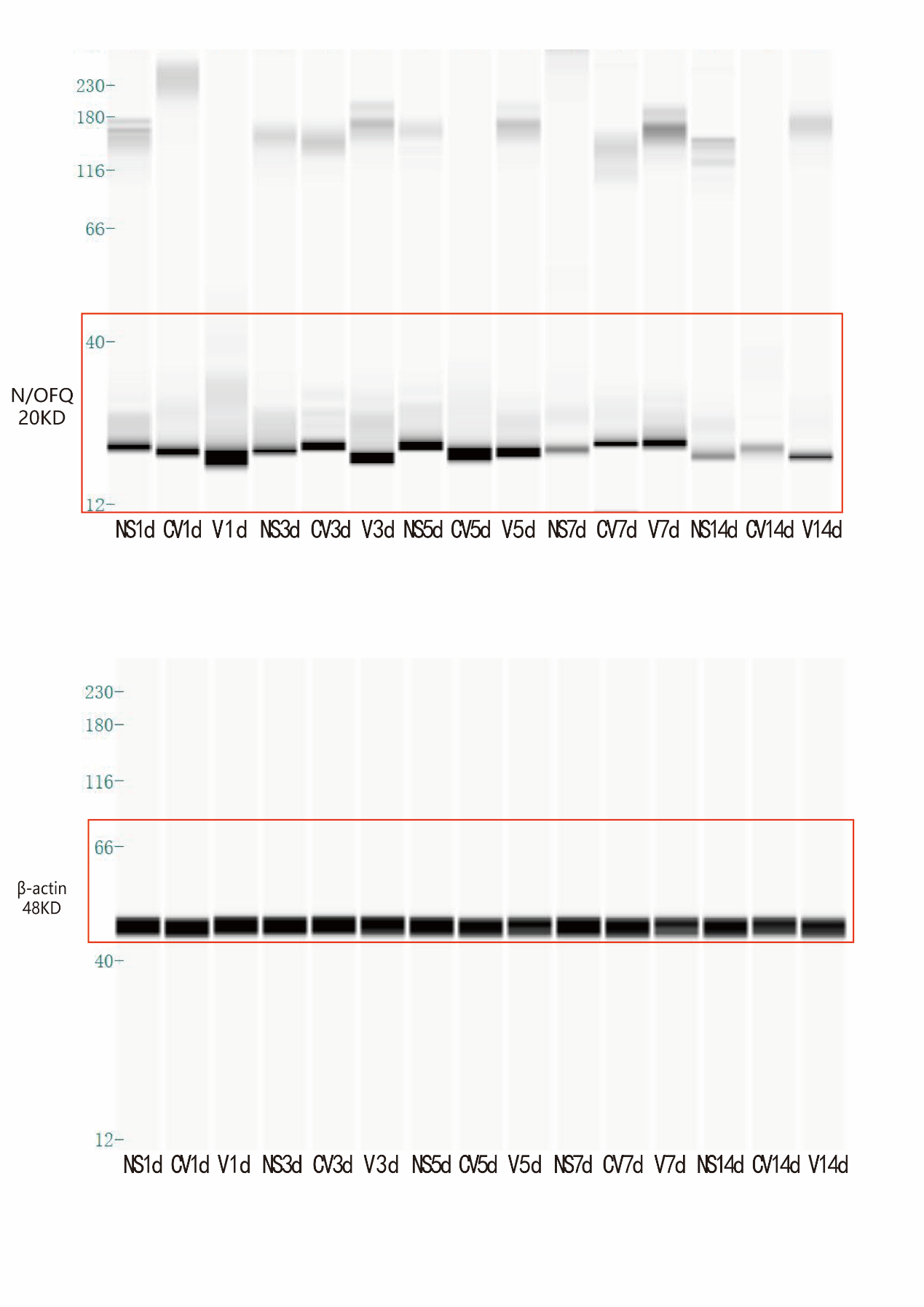

Supplement: Supplementary file 3 — Additional file 3: Figure S1. Full results of Western blot analysis for the quantification of N/OFQ expression in trigeminal ganglia. Figure S2. Full results of Western blot analysis of CGRP expression in trigeminal ganglion of rats in NS group, N/OFQ group and UFP-101 group. Figure S3. Full results of Western blot analysis of N/OFQ expression in trigeminal ganglion of rats in NS group, control lentivirus group and PNOC lentivirus group. [file 12868_2021_632_MOESM3_ESM.docx]
